# Multiple paraneoplastic syndromes revealing non-small cell lung carcinoma

**DOI:** 10.11604/pamj.2014.19.237.4409

**Published:** 2014-11-03

**Authors:** Monia Youssef, Melek Kechida, Saoussen Cheikhmohamed, Adnene Moussa, Jameleddine

**Affiliations:** 1 Dermatology Departement, Fattouma Bourguiba Hospital Monastir, Tunisia; 2 Pneumology Departement, Fattouma Bourguiba Hospital Monastir, Tunisia; 3 Anatomo-Pathology Departement, Fattouma Bourguiba Hospital Monastir, Tunisia

**Keywords:** Carcinoma, paraneoplastic dermatoses, IgE

## Abstract

A broad spectrum of paraneoplastic dermatoses is associated with lung cancer. We report herein a 56-year-old man who presented an association of erythroderma, acquired ichthyosis, palmo-plantar keratoderma, hypereosinophilia and hyper IgE. In light of these clinical and biological assessments an underlying malignancy had been suspected. A thoracic, abdominal and pelvic computed tomography showed a left hilar mass. The patient underwent a left pneumonectomy and the histological study had confirmed a non-small cell lung cancer. Recognition of cutaneous paraneoplastic syndromes is important since it leads to prompt diagnosis of an underlying malignancy and consequently a better management and prognosis of the disease.

## Introduction

Cutaneous paraneoplastic syndromes are a large group of dermatoses that may be associated with an internal malignancy. A broad spectrum of paraneoplastic dermatoses is associated with lung cancer. We report herein the case of patient who presented an association of erythroderma, acquired ichthyosis, palmo-plantar keratoderma, hypereosinophilia and hyper IgE that revealed a non-small-cell lung carcinoma (NSCLC).

## Patient and observation

**Figure 1:**
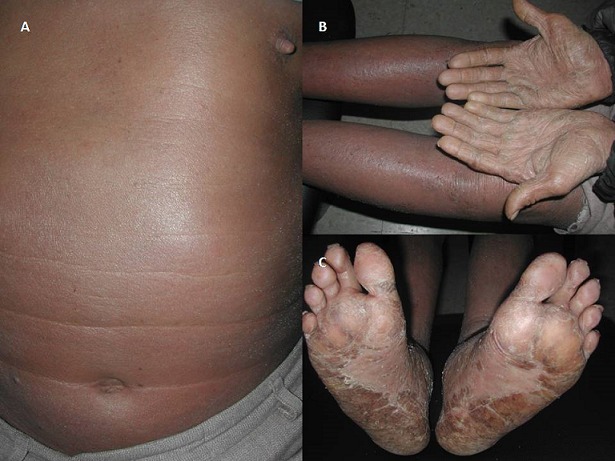
(A) erythematous and finely scaling lesions on the trunk; (B, C) palmo-plantar keratoderma

**Figure 2:**
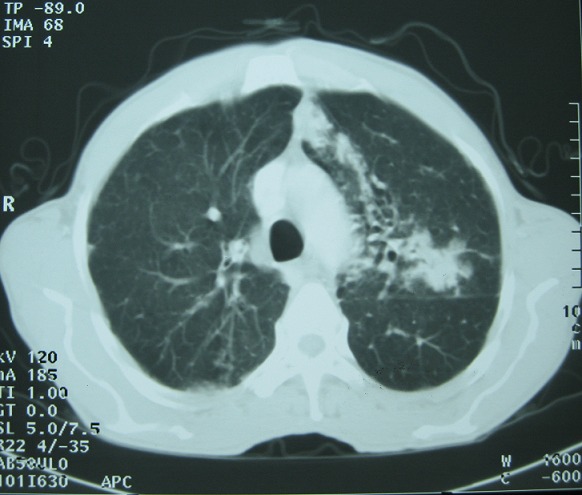
The scanographic aspect of the left hilar mass

A 56-year-old man was admitted to our dermatology department on November 2011 for the onset of a three-month history of erythroderma associated with weight loss, asthenia and chronic diarrhea. He was a heavy smoker, with no past medical history. The physical examination on admission revealed generalized itchy, erythematous and scaling lesions on the face, trunk, and extremities predominantly on the extensor surfaces, associated with palmo-plantar keratoderma (Figure 1). The biological assessment revealed a biological inflammatory syndrome with an accelerated erythrocyte sedimentation rate at 85 mm, hypergammaglobulinemia at 16g/l, inflammatory anemia at 8.9 g/dl with hypereosinophilia ranging from 2500 to 3600 E/mm^3^ and hyper IgE serum level reaching 13932 U/ml. Cutaneous biopsies showed focal acanthosis and focal parakeratosis, hypogranulosis, with mild dermal perivascular infiltrate consisting mainly of lymphocytes and some eosinophils. Tumor markers showed only mildly elevated carcinoembryogenic antigen (CEA). A thoracic, abdominal and pelvic computed tomography showed a left hilar mass of 6 cm with micronodules of the left upper lobe without any other metastatic lesions (Figure 2). A stenotic infiltration of the culmen was found on the bronchial fibroscopy. The biopsy of this infiltration showed a bronchogenic epidermoid carcinoma. This diagnosis was confirmed by the histological analysis when the patient underwent a left pneumonectomy. The clinical course was characterized by a complete healing of the cutaneous lesions and a regression of the hyper eosinophilia and IgE. One year later the patient died due to the recurrence of his cancer.

## Discussion

Our case highlights the association of multiple paraneoplastic syndromes: transient erythroderma, acquired ichthyosis, palmo-plantar keratoderma, hyper eosinophilia and hyper IgE. The parallel course of the carcinoma, the cutaneous lesions and these hyper IgE and eosinophilia strongly supports paraneoplastic phenomenon. Erythroderma may be a cutaneous manifestation of malignancy. Its association with internal malignancy is approximately 1% [1]. Acquired ichthyosis (AI) is associated with malignancy in nearly half of the cases. One-third of the reported cancers were related to Hodgkin′s disease. Reported solid tumors associated with AI are breast, bronchial, laryngeal, esophageal, gastric bladder, transitional cell carcinoma of kidney, cervix and ovarian cancer [2].

Association of AI to other cutaneous paraneoplastic syndromes is rare. Some isolated cases of association have been reported in literature with erythroderma that evolved to erythema gyratum repens [3], bazex paraneoplastic acrokeratosis, addisonian pigmentation and dermatomyositis [4]. Association of lung carcinoma with paraneoplastic cutaneous manifestations has been widely reported in literature. A Pub Med literature search on cutaneous paraneoplastic manifestations associated with NSCLC from 1970 to February 2014 showed that there is no specific paraneoplastic dermatosis associated with this type of lung cancer. This latter is most commonly associated with neurologic, rheumatic and endocrine paraneoplastic syndromes. Clubbing and hypertrophic osteoarthropathy occurs in 1-5% of all patients with NSCLC and is considered as the most reported paraneoplastic dermatosis [5, 6] but it is not specific to NSCLC.

## Conclusion

We consider our case to be original because, to our knowledge, it is the first report in literature invoking the association of acquired ichthyosis with NSCLC, and also reporting a myriad of paraneoplastic cutaneous and hematologic syndromes revealing a NSCLC.
